# Assessment of risk factors for delayed gastric emptying after distal gastrectomy for gastric cancer

**DOI:** 10.1038/s41598-022-20151-5

**Published:** 2022-09-23

**Authors:** Tomosuke Mukoyama, Shingo Kanaji, Ryuichiro Sawada, Hitoshi Harada, Naoki Urakawa, Hironobu Goto, Hiroshi Hasegawa, Kimihiro Yamashita, Takeru Matsuda, Taro Oshikiri, Yoshihiro Kakeji

**Affiliations:** Division of Gastrointestinal Surgery, Department of Surgery, Kobe University Graduate School of Medicine, 7-5-2, Kusunoki-cho, Chuo-ku, Kobe, Hyogo Japan

**Keywords:** Gastroenterology, Gastrointestinal diseases, Stomach diseases, Gastric cancer

## Abstract

The risk factors for delayed gastric emptying (DGE) following gastrectomy are unclear. This study aimed to investigate the risk factors for DGE and the severity of DGE. We retrospectively evaluated 412 patients who underwent gastrectomy for gastric cancer between 2011 and 2019. The cases were classified into the DGE (n = 27) and non-DGE (n = 385) groups; the DGE group was further classified into two subgroups based on nasogastric tube insertion as an indicator of severity. For determining the relationship between resected stomach volume and DGE, we calculated the area of each surgical specimen using the ImageJ software. Female sex (odds ratio [OR] 2.55; 95% confidence interval [CI] 1.09–5.93; *P* = 0.03), diabetes (OR 2.38; 95% CI 1.02–5.57; *P* = 0.03), and distal gastric tumors (OR 2.61; 95% CI 1.10–6.19; *P* = 0.02) were identified as independent risk factors by multivariate analysis. The duration of hospital stay was longer in the DGE group than in the non-DGE group (29 vs. 15 days, *P* < 0.01). Overall, 24 cases of DGE (89%) were found in more than 1 week following surgery. No correlation was observed between clinical features and the severity of DGE. The resected area in the DGE group was significantly larger than that in the non-DGE group (198.0 vs. 173.9 cm^2^, *P* = 0.03). In conclusion, DGE was frequently observed in females and in patients with diabetes and distal gastric tumors. Most of the DGE cases occurred after 7–14 days of surgery, patients who are discharged early should be informed to seek hospitalization if they have symptoms caused by DGE.

## Introduction

Globally, gastric cancer is the fifth most common cancer and the third most common cause of death. Although minimally invasive surgical procedures have recently become increasingly popular, complications still occur. Delayed gastric emptying (DGE) represents one of the major complications following gastrectomy for gastric cancer, with an incidence of approximately 5–25%^1^. Most previous studies have focused on DGE following pancreaticoduodenectomy, failing to elucidate the mechanism for DGE after distal gastrectomy. DGE is characterized by the stasis of gastric contents, manifesting with symptoms, such as epigastric fullness, anorexia, nausea, and vomiting. Generally, it is not a fatal condition and can be treated with either nasogastric tube (NGT) insertion into the remnant stomach or simply fasting with or without prokinetic therapy. Nevertheless, it delays oral intake and leads to prolonged hospital stay and sometimes to a significant decrease in patients’ quality of life.

Some studies have suggested that the innervation of the remnant stomach by the dissected vagus nerve contributes to DGE^2,3^. Furthermore, 10–30% of patients undergoing Roux-en-Y (RY) reconstruction have been reported to develop DGE, which is known as the Roux stasis syndrome (RSS)^4^. A recent study in Japan failed to show the noninferiority of retrocolic gastrointestinal (GI) reconstruction to antecolic reconstruction regarding the incidence of DGE following pancreaticoduodenectomy (PD), thereby suggesting that the digestive tract reconstruction should not be performed via the retrocolic route^5^. Although some studies have demonstrated that a relatively large remnant stomach affects DGE^6^, none of them have provided any data on the remnant stomach volume. Other than surgical techniques, multiple comorbidities, such as diabetes mellitus and obesity, could also be the underlying causes of DGE^7^.

Although numerous studies have attempted to explain the mechanism of DGE, it remains unclear. Here we retrospectively evaluated the risk factors for DGE and investigated the relationship between the volume of the remnant stomach and the incidence of DGE by calculating the area of the resected stomach on macroscopic images.

## Materials and methods

### Study design

In total, 412 patients who underwent distal gastrectomy for gastric cancer at Kobe University Hospital between April 2011 and December 2019 were included in the study. Subsequently, they were classified into two groups: DGE (n = 27) and non-DGE (n = 385). Clinicopathological, demographic, and perioperative data were retrospectively collected from a database of our hospital. The study was approved by the research ethics committee of Kobe University Hospital (No. B210054). All methods were conducted in accordance with the relevant guidelines and regulations of the committee. All patients provided written informed consent for the anonymous use of surgical data.

### Surgical procedures

D1 plus or D2 lymphadenectomy was performed based on the Japanese gastric cancer treatment guidelines^8^. The surgical procedures included Billroth I, Billroth II, or RY reconstruction and were selected at the discretion of individual surgeons. RY gastrojejunostomy was performed either laparoscopically via the antecolic route or by laparotomy via the retrocolic route, with a stapler used in both procedures. The jejunal anastomosis was placed 35 cm distal to the gastrojejunal anastomosis.

### Definition of DGE

We defined DGE as (1) clinical symptoms, such as epigastric fullness, nausea, and vomiting, and (2) over 7 days of continuous fasting following gastrectomy, or re-fasting^2^. The definition of DGE proposed by the International Study Group of Pancreatic Surgery was not used because it only defined cases requiring NGT for the treatment as DGE. Instead, a different definition was used that included cases manifesting with DGE symptoms and requiring fasting but not NGT. After confirmation of stomach distension by plain abdominal X-ray scan, some patients underwent an upper GI series or computed tomography. Some patients with DGE underwent GI endoscopy to rule out anastomotic stenosis and were excluded from the DGE group if any mechanical obstruction was detected. The DGE group was further categorized into two subgroups, namely, NGT and non-NGT, depending on NGT insertion as an indicator of symptom severity; that is, prokinetic therapy was sometimes required in the NGT group even after NGT removal, whereas patients in the non-NGT group recovered only by fasting or taking prokinetics. Patients were allowed to drink water on postoperative days (PODs) 1–3 and usually started eating solid foods on PODs 3–4. Attending surgeons visited the patients and prescribed fasting or re-fasting depending on their symptoms.

### Resected area calculation

We hypothesized that distal gastric tumors allowed us to resect less volume than proximal tumors, thereby leaving a larger remnant stomach. Therefore, the volume of the resected stomach, which in turn was deemed to approximate the area of the resected specimen, is associated with the incidence of DGE.

**Figure 1:**
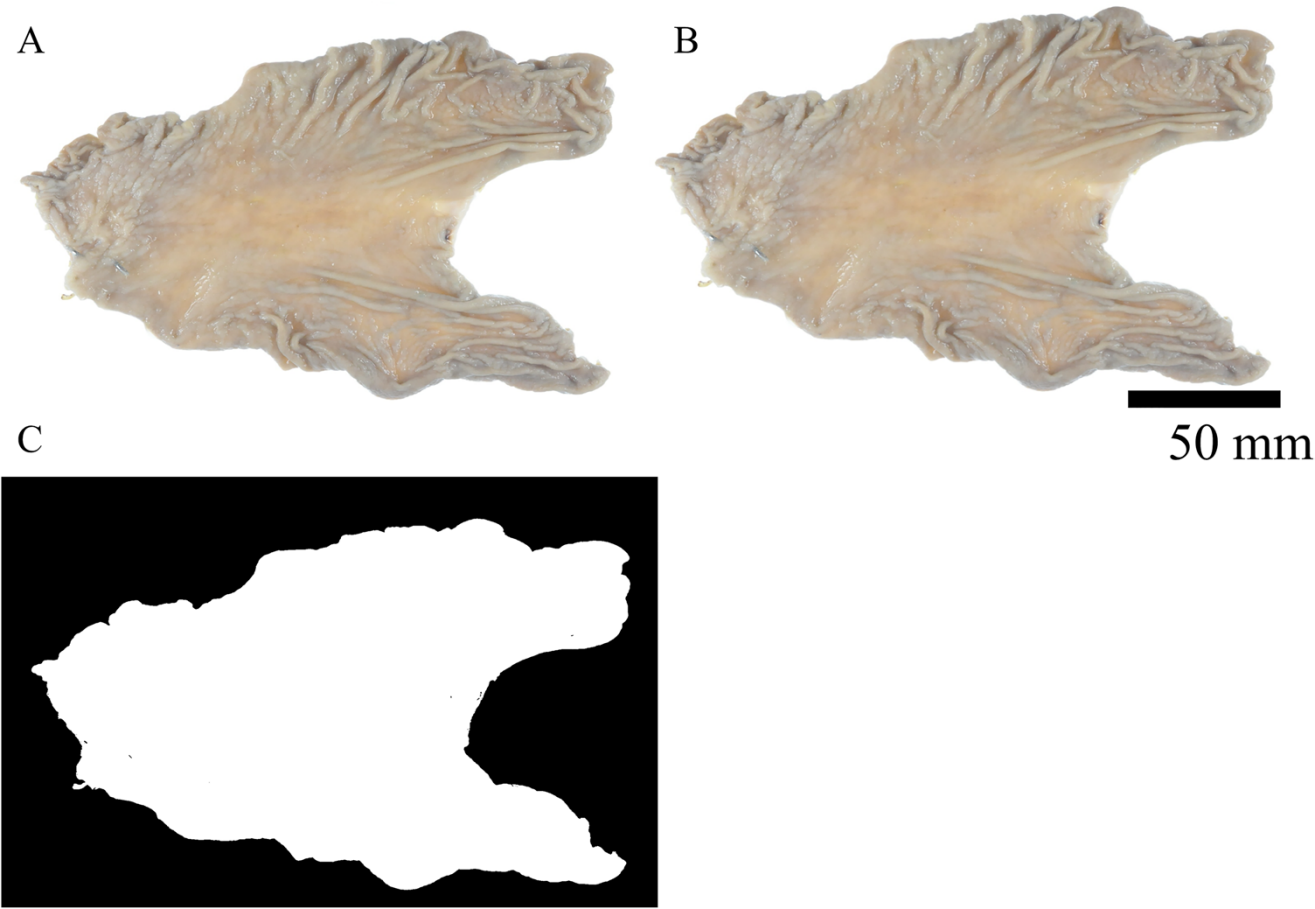
Resected area calculation using ImageJ: (**A**) Import the original image to ImageJ. (**B**) Set the scale bar along the ruler in the original image and define its length as 50 mm. (**C**) Process the image to make it binary. Calculate the white area and output each result into Excel. Subsequently, extract the largest value and define it as the resected area.

To calculate the area of the resected stomach, we collected macroscopic images of surgical specimens from patient clinical records and analyzed them using ImageJ^9^, which is a free image-processing software. The calculation process is shown in detail in Fig. 1.

### Statistical analysis

Patient data were retrieved from electronically stored medical records using Microsoft Excel (Microsoft, Redmond, WA). Continuous variables were expressed as median and range, and categorical variables were expressed as frequency and percentage. Continuous data were analyzed with the t-test and Mann–Whitney U test, whereas categorical data were compared using the chi-square test, Fisher’s exact test, and Kruskal–Wallis test. The variables for which *P* values were less than 0.2 in univariate analysis were included in multivariate analysis. We determined the cutoff value of the continuous variables as the maximum Youden index value determined by receiver operating characteristic (ROC) curve analysis. A *P* value of < 0.05 was considered statistically significant. All statistical analyses were conducted with EZR^10^, which is a modified version of R Commander designed to add statistical functions frequently used in biostatistics.

## Results

The overall incidence of DGE was found to be 6.6% (27 of 412 patients). The median day of onset was POD 10 (range, 7–12 days), with a median time to recovery of 10 (range 5–14) days. NGT insertion was necessitated in 12 cases (NGT group).

The clinical and surgical characteristics of patients in each group are presented in Table 1. There were no significant differences between the groups in terms of sex, age, body mass index (BMI), American Society of Anesthesiologists Physical Status score, and tumor–node–metastasis classification.

**Table 1:** Demographic and surgical characteristics of patients.

	DGE (n = 27)	non-DGE (n = 385)	*P* value
**Sex**			0.13
Male	12 (44.4%)	268 (69.6%)	
Female	15 (55.6%)	117 (30.1%)	
**Age median [IQR]**	75.0 [70, 78]	71 [65, 77]	0.09
**BMI**	22.9 [21.0,25.7]	22.3 [20.2, 24.3]	0.21
> 25	8 (29.6%)	74 (13.8%)	
< 25	19 (70.4%)	310 (86.2%)	
**ASA PS classification**			0.098
< 3	20 (5.7%)	332 (94.3%)	
≥ 3	7 (11.7%)	53 (88.3%)	
**Diabetes**			0.026
Yes	11 (40.7%)	78 (20.3%)	
No	16 (59.3%)	306 (79.7%)	
**Location of tumor**			0.033
Proximal	0 (0%)	14 (3.6%)	
Middle	9 (33.3%)	215 (55.8%)	
Distal	18 (66.7%)	156 (40.6%)	
**T stage**			0.84
T1	18 (66.7%)	245 (63.6%)	
≥ T2	9 (33.3%)	141 (36.4%)	
**N stage**			0.83
N0	18 (66.7%)	266 (69.0%)	
N ≥ 1	9 (33.3%)	119 (31%)	
**M stage**			> 1
M0	26 (96.3%)	372 (96.6%)	
M1	1 (3.7%)	13 (3.4%)	
**TNM stage**			0.41
I	18 (66.7%)	258 (67.0%)	
II	2 (7.4%)	63 (16.4%)	
III	6 (22.2%)	49 (12.7%)	
IV	1 (3.7%)	13 (3.9%)	
**Operation time (minutes) median [IQR]**	315 [284.5, 351.0]	293 [238.5, 360.5]	0.09
**Blood loss (ml) median [IQR]**	75 [0, 387.5]	7[0, 150]	0.056
**Approach**			0.14
Laparotomy or conversion	9 (33.3%)	78 (20.2%) ara>	
Laparoscopy	18 (66.7%)	307 (79.8%)	
**Lymph node dissection**			0.44
D1+	14 (51.9%)	217 (56.4%)	
D2	13 (48.1%)	145 (37.7%)	
D2+	0 (0%)	21 (5.9%)	
**Reconstruction**			0.66
B-I	14 (51.9%)	222 (57.7%)	
R-Y	13 (48.1%)	159 (41.3%)	
Other	0 (0%)	4 (1.0%)	
**Length of hospital stay (days) median [IQR]**	29 [25, 34.5]	15 [7, 21]	< 0.01

*IQR* interquartile range, *DGE* delayed gastric emptying.

The incidence of DGE was significantly higher in patients with diabetes (*P* = 0.026) and distal gastric tumors (*P* = 0.033). The groups demonstrated no significant difference in operation time, blood loss, surgical procedure, lymphadenectomy extent, and reconstruction type. As presented in Table 2, the area of the resected stomach was calculated to be significantly larger in the DGE group than in the non-DGE group (198.0 cm^2^ vs. 173.9 cm^2^, respectively; *P* = 0.03). Furthermore, the length of hospital stay was significantly longer in the DGE group than in the non-DGE group (29 days vs. 15 days, respectively; *P* < 0.01).

**Table 2:** Calculation of the resected stomach area using ImageJ.

	DGE	Non-DGE	*P* value
Resected area (cm^2^) median [IQR]	198 [165.3, 232.0]	173.93 [147.4, 205.3]	0.03

*IQR* interquartile range, *DGE* delayed gastric emptying.

Univariate analysis revealed that DGE was significantly associated with diabetes, distal gastric tumors and the area of the resected stomach. Sex, age, operation time, blood loss, and surgical approach were entered as independent risk factors for DGE along with diabetes and distal gastric tumors in a multivariate logistic regression model, where female sex, diabetes and distal gastric tumors were identified as independent risk factors for DGE (Table 3).

**Table 3:** Univariate and multivariate analyses of risk factors associated with delayed gastric emptying.

	Univariate analysis			Multivariate analysis		
	OR	*P* value	95% CI	OR	*P* value	95% CI
Female	1.83	0.13	0.83–4.04	2.47	0.037	1.05–5.79
Age > 73 years	2.08	0.075	0.93–4.67	2.15	0.078	0.92–5.04
Diabetic	2.7	0.016	1.20–6.04	2.44	0.041	1.04–5.73
Tumors in the distal one-third portion	2.94	0.01	1.29–6.70	2.59	0.033	1.08–6.19
Operation time ≥ 261 min	3.94	0.03	1.16–13.3	3.32	0.061	0.94–11.7
Blood loss ≥ 251 ml	2.80	0.01	1.25–6.27	2.49	0.12	0.80–7.78
Laparotomy	1.97	0.11	0.85–4.55	0.62	0.45	0.19–2.11
Resected area ≥ 189.9 cm^2^	2.36	0.03	1.07–5.18	2.06	0.11	0.86–4.93

The detailed clinical characteristics of patients in the DGE group are presented in Table 4. No correlation was observed between the severity of DGE and the clinical features—such as female sex, diabetes, and distal gastric tumors—that were identified as risk factors for DGE. The duration until discharge was significantly longer in the NGT group than in the non-NGT subgroup (23 days vs. 20 days, respectively; *P* = 0.04). The results revealed that 24 cases (89%) of DGE were observed after > 1 week of discharge.

**Table 4:** Severity and clinical features of delayed gastric emptying.

	NGT insertion (n = 12)	NGT non-insertion (n = 15)	*P* value
**Sex**			1
Male	7 (58.3%)	3 (53.3%)	
Female	5 (41.7%)	7 (46.4%)	
**Diabetes**	6 (50%)	5 (33.3%)	0.45
**Tumors in the distal one-third portion**	9 (75.0%)	9 (60.0%)	0.68
**Prokinetic drugs use**	7 (58.3%)	4 (26.7%)	0.13
**Duration to onset (days)**			0.14
< 7	3 (25%)	0 (0%)	
< 7–14	8 (66.7%)	12 (80%)	
> 14	1 (8.3%)	3 (20%)	
**Duration to recovery (days from onset) median [IQR]**	10.5 [8.75, 12.5]	6.0 [2.5, 14.0]	0.2
**Duration to discharge (days from onset) median [IQR]**	23 [19.8, 30.5]	20 [16.0, 22.0]	0.04

*NGT* nasogastric tube, *IQR* interquartile range.

## Discussion

In this study, we demonstrated that female sex, distal gastric tumors, and diabetes were the risk factors for DGE. The DGE incidence of 6.6% found in this study was comparable to 5–25% reported in a previous study^1^. However, the incidence of DGE found in this study was low at 3.0% when it was limited to cases that required NGT therapy, which is the criterion of DGE after pancreaticoduodenectomy according to an international study group of pancreatic fistula^11^. Patients with DGE requiring NGT insertion stayed longer in the hospital, which reflects the fact that they had more severe conditions compared with those in the non-NGT subgroup. In all cases except three, DGE occurred after > 7 days postoperatively, indicating that patients who are discharged early still need careful evaluation at subsequent outpatient visits. In these cases, physicians should always keep in mind the possibility of DGE development after hospital discharge and therefore continue dietary support.

The mechanism that female contributes to DGE remains unclear; however, several studies have specified the gastric emptying abnormalities are more frequent in female than male patients^12^. Premenopausal women tend to show impaired gastric motility owing to increased estrogen and progesterone levels. Moreover, postmenopausal women who take hormone replacement with estrogen and progesterone also show slower gastric emptying of solids^13^. These differences in sex hormones can explain the higher incidence of DGE in females.

Several studies have suggested that distal gastric tumors contribute to the development of DGE. One of the proposed hypotheses is that when the remnant stomach is larger, it tends to become more atonic and consequently more prone to DGE as the routinely dissected vagus nerve has to innervate a larger anatomical region^2^. Although the area of the resected stomach did not remain an independent risk factor for DGE, to the best of our knowledge, the present study is the first to assess the resected volumes of the stomach. The resected area was found to be significantly larger in the DGE group than in the non-DGE group. This result contradicts our hypothesis that the resected volume is smaller in distal gastric tumors. However, as we routinely resect two-thirds of the stomach in distal gastrectomy regardless of tumor location, the proportion of resected stomach size to whole stomach size was considered in this study. Therefore, the remnant stomach may be large in cases where the resected stomach is proportionally large. Moreover, we compared the resected area between the DGE and non-DGE groups in R-Y and B-I reconstructions, respectively. For both the R-Y and B-I subgroups, the resected area tended to be larger in the DGE group than that in the non-DGE group (R-Y subgroup: 210.2 vs. 188.1 cm^2^; *P* = 0.45 and B-I subgroup: 153.8 vs. 140.4 cm^2^; *P* = 0.19). Consequently, we believe that a larger resected stomach size correlates with a larger remnant stomach, which contributes to the incidence of DGE. However, neither the present nor previous studies have shown that the size or volume of the resected stomach and the remnant stomach are proportional based on computed tomography (CT) images. Therefore, it is difficult to compare the size of the remnant stomach using CT images because of interpersonal differences as well as differences in the degree of gastric distention and the amount of residue in fasting patients. Huh et al. administered an effervescent agent in patients before performing CT scan to assess the remnant stomach volume^14^. The retrospective nature of the present study is an important limitation. To determine the relationship between DGE and the stomach remnant volume, a prospective study calculating the remnant stomach volume with CT images using an effervescent agent preparation is required.

Diabetes mellitus is another risk factor that remained significant in multivariate analysis. A recent study reported that 9.8% of patients with type 1 diabetes experienced symptoms of gastroparesis. It has also been reported that patients with either type 1 or type 2 diabetes tend to suffer these symptoms more often than those without diabetes^15,16^. One of the suggested mechanisms is that acute hyperglycemia slows gastric emptying by suppressing antral contractions and stimulating pyloric contractions^17^. Since it is difficult to achieve strict glycemic control in the postsurgical period due to unstable food intake and continuous intravenous infusion, some patients develop a hyperglycemic condition, which in turn may lead to DGE. Mao et al. reported that preoperative hyperglycemia, rather than hemoglobin A1c levels, could effectively predict DGE following subtotal gastrectomy and presented a model that incorporated multiple risk factors to predict postsurgical gastroparesis^18^. Considering the physiological relationship between hyperglycemia and DGE, such a predictive model can prove especially useful. Moreover, stricter postsurgical glycemic control should be achieved by diabetes physicians in patients at a high risk for DGE. As a limitation, our study did not include data on perioperative levels of hemoglobin A1c or blood glucose; such data could have enabled further investigation of the relationship between diabetes and DGE.

At our hospital, Billroth I reconstruction is generally used as the first choice of treatment for distal gastric cancer. However, surgeons tend to perform an RY reconstruction when the remnant stomach is too small for a Billroth I reconstruction. We believe that RY reconstruction with a small remnant stomach helps reduce the incidence of DGE. However, in contrast, the incidence of functional DGE (also known as RSS) following RY reconstruction is reportedly higher than that after Billroth I reconstruction^18,19^. Many causes of RSS have been proposed, including ectopic pacemakers originating in the Roux limb, driving contractions in a reverse direction, and gastroparesis of the remnant stomach after vagotomy^20–22^. When the incidence of DGE was limited to RY reconstruction in the present study, it was found to be lower than the previous study’s incidence of DGE, which ranged from 10 to 30%^2^. In this study, we hypothesized that a smaller remnant stomach following RY reconstruction would reduce the potential risk of RSS after RY reconstruction. Hence, this fact may have influenced the low incidence of DGE in our study.

Furthermore, pancreatic fistula is often associated with DGE. This is supported by the high incidence of DGE (19–57%) following PD. Pancreatic fistula continues to be a major complication of PD that, in severe cases, necessitates prolonged drainage tube placement. Local inflammations and intra-abdominal abscesses impair GI motility. In the current study, the overall incidence of pancreatic fistula was 6.6%, which had no association with DGE. This is because pancreatic fistula following distal gastrectomy is mostly a minor leakage that does not impair gastric emptying and can be treated with oral or intravenous antibiotics. Thus, the association between DGE and pancreatic fistula after gastrectomy is not as strong as that after PD.

In conclusion, this study demonstrated that the female sex, distal gastric tumors, and diabetes were the risk factors for DGE after gastrectomy for gastric cancer. As most DGE cases occurred 7–14 days after surgery, patients with DGE who were discharged early after surgery without symptoms should be carefully informed about the major signs of DGE and should seek hospitalization if they have symptoms caused by DGE.

## Data Availability

All data generated or analyzed during this study are included in this published article.
